# Obesity and obstructive sleep apnea: from pathophysiology to current clinical approaches

**DOI:** 10.36416/1806-3756/e20250314

**Published:** 2026-08-13

**Authors:** Erika Cristine Treptow, Glaucia Carneiro, Monica Levy Andersen, Luciano F Drager, Pedro Rodrigues Genta

**Affiliations:** 1. Departamento de Psicobiologia, Universidade Federal de São Paulo, São Paulo (SP) Brasil.; 2. Instituto do Sono, Associação Fundo de Incentivo à Pesquisa - AFIP - São Paulo (SP) Brasil.; 3. Divisão de Endocrinologia, Departamento de Medicina, Universidade Federal de São Paulo, São Paulo (SP) Brasil.; 4. Unidade de Hipertensão, Instituto do Coração - InCor - Hospital das Clínicas, Faculdade de Medicina, Universidade de São Paulo - HCFMUSP - São Paulo (SP) Brasil.; 5. Unidade de Hipertensão, Disciplina de Nefrologia, Hospital das Clínicas, Faculdade de Medicina, Universidade de São Paulo - HCFMUSP - São Paulo (SP) Brasil.; 6. Unidade de Distúrbios do Sono, Divisão de Pneumologia, Instituto do Coração - InCor - Hospital das Clínicas, Faculdade de Medicina, Universidade de São Paulo - HCFMUSP - São Paulo (SP) Brasil.

**Keywords:** Obesity, Sleep apnea, obstructive, Obesity management

## Abstract

The prevalence of obesity and obstructive sleep apnea (OSA) has increased alarmingly over recent decades and is projected to continue to rise. These chronic disorders interact bidirectionally and increase cardiovascular and metabolic risks. OSA is a complex and heterogenous disease in which weight management plays a key role in achieving long-lasting therapeutic success. Multiple mechanisms are associated with the link between obesity and OSA, including enlargement of upper airway soft tissue, pharyngeal lengthening, reduced lung volume, and leptin impairment. In this review, we describe the mechanisms linking obesity and OSA, and examine the impact of CPAP therapy on body weight. We also review current obesity treatment strategies and their effects on reducing OSA severity, comparing the efficacy of lifestyle modifications, surgical approaches, and pharmacological treatments in promoting weight loss. Finally, we explore the potential of recent advances in pharmacological obesity treatments as therapeutic options for OSA, as well as the promise of combination therapy for achieving better short- and long-term weight reduction.

## INTRODUCTION

Obesity is a chronic, multifactorial disease that has increased worldwide in recent decades.^1^ In 2021, an estimated 1.00 billion adult males (95% CI, 0.989-1.01) and 1.11 billion adult females (95% CI, 1.10-1.12) worldwide were living with overweight or obesity.^2^ Similarly, obstructive sleep apnea (OSA) is estimated to affect 936 million adults in the 30- to 69-year age bracket worldwide.^3^ Obesity and OSA have been increasingly recognized as interconnected disorders, each exacerbating the other in a vicious cycle. There is ongoing debate about whether obesity or sleep disorders come first.^4^ However, it is undeniable that they influence each other bidirectionally.^4^ Obese individuals often develop sleep disorders, particularly respiratory disturbances such as OSA and obesity hypoventilation syndrome, while sleep disorders can contribute to weight gain and difficulties in weight loss.^5^

Obesity and overweight are considered the most significant risk factors for the development of OSA. In a population-based study conducted in Brazil, it was reported that being overweight was associated with 2.6-fold higher odds of developing OSA, and a BMI > 30 kg/m^2^ increased the odds by 10.5-fold.^6^ Both obesity and OSA may lead to the development and aggravation of cardiovascular and metabolic diseases through oxidative stress, sympathetic activation, endothelial dysfunction, and chronic low-grade inflammation.^7^

Obesity is a modifiable risk factor for OSA, and weight management is a fundamental part of OSA treatment.^8^ In this review, we address the relationship between OSA and obesity, and explore weight loss interventions and their potential for improving breathing during sleep. 

## PATHOPHYSIOLOGY OF OBESITY AND OSA

**Figure 1:**
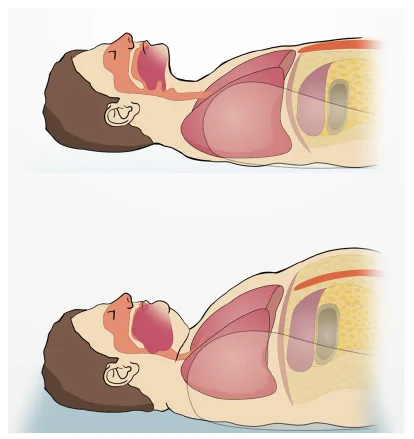
Effect of obesity on the upper airway. In A, schematic representation of the upper airway of an individual with normal weight and without obstructive sleep apnea (OSA). In B, upper airway of an obese individual with OSA. In comparison with the normal-weight individual, the obese individual has an enlarged tongue as a result of fat infiltration. The enlarged tongue may lengthen the upper airway and narrow the pharynx. Another effect of obesity is a reduction in the tracheal tug on the upper airway as a result of reduced lung volume (see text for details).

Anatomical and humoral mechanisms are involved in the association between obesity and OSA (Figure 1). Obesity may lead to enlargement of upper airway soft tissue, pharyngeal lengthening,^9^ reduced lung volume, reduced tracheal tug, and leptin resistance. 

While overweight and obesity are associated with upper airway soft tissue enlargement, weight loss reduces the anatomical impairments associated with OSA.^10^ ^,^ ^11^ Fat infiltration of upper airway muscles is thought to underlie this tissue enlargement,^10^ ^,^ ^12^ which increases upper airway collapsibility and, consequently, the propensity to OSA.^13^ In a 2014 study comparing obese individuals with and without OSA, it was found that those with OSA had enlarged tongue volumes and increased fat within the tongue when compared with controls.^14^ Increased tongue volume is associated with narrowing of the upper airway in the retroglossal region. More recently, a study conducted in 2020 showed that OSA improvement following weight loss after intensive lifestyle modification or bariatric surgery was mediated by reductions in tongue fat .^11^

Pharyngeal lengthening is another mechanism involved in the link between obesity and OSA. The anatomical landmarks of pharyngeal length are the posterior nasal spine and the origin of the epiglottis. Obesity can cause an increase in pharyngeal length by tongue enlargement, resulting in caudal hyoid displacement.^9^ ^,^ ^12^ Given that the hyoid bone is attached to the epiglottis, tongue enlargement and caudal hyoid displacement result in pharyngeal lengthening. An increased pharyngeal length may worsen pharyngeal collapsibility and OSA severity ^9^

Obesity also impairs lung volume and function. In comparison with peripheral adiposity, abdominal adiposity is more strongly associated with reductions in lung volume and respiratory function.^15^ The pharynx is connected to the trachea by mediastinal connective tissue, and upper airway dimensions are closely linked to lung volume.^16^ As the lungs inflate from residual volume to total lung capacity, the upper airway progressively enlarges. This occurs because higher lung volume stiffens the pharynx through the downward movement of the trachea, a mechanism known as tracheal tug. Conversely, reduced lung volume decreases this traction, weakening the pharyngeal walls and making them more compliant to collapse. Therefore, obesity-related reductions in lung volume further promote upper airway narrowing. The independent impact of lung volume impairment in the pathogenesis of OSA is supported by studies showing that increased lung volume induced by applying negative pressure around the chest reduces upper airway collapsibility and OSA severity.^16^ ^-^ ^18^

While reduced lung volume represents a mechanical pathway through which obesity promotes upper airway collapse, obesity-related hormonal disturbances provide an additional, distinct mechanism. 

Leptin is an adipocyte-derived hormone, and its levels are associated with the level of adiposity. Its primary role is to regulate energy balance by inhibiting hunger through hypothalamic networks. It also regulates ventilatory function through the brain and peripheral nervous system via carotid body chemoreflex. In animal models, leptin deficiency results in blunted hypercapnic and hypoxemic ventilatory responses and increases upper airway resistance and collapsibility. In humans, a study of predominantly class III obese women with OSA found that higher leptin levels were associated with increased upper airway muscle recruitment during airflow limitation.^19^ Paradoxically, while leptin concentrations rise proportionally with adiposity, obesity simultaneously attenuates the physiological actions of leptin through central leptin resistance and saturation of its blood-brain barrier transport mechanisms.^20^ Together, these findings suggest that leptin deficiency or resistance plays a role in the pathogenesis of OSA. 

## LIFESTYLE INTERVENTIONS

Regardless of OSA severity, lifestyle interventions such as diet and exercise should be considered for all overweight and obese patients. Such interventions can be applied in isolation or in combination with specific OSA treatments to improve outcomes.^21^ However, lifestyle advice alone is often insufficient to prompt meaningful weight loss in patients with OSA. The 2018 Ofﬁcial American Thoracic Society Clinical Practice Guideline highlighted that the discussion of weight loss management and lifestyle interventions with OSA patients during consultations is insufficient to achieve the best results.^8^ Obese patients are more likely to initiate a weight loss program if they are referred for a speciﬁc program rather than simply being advised to lose weight.^8^

One of the most effective strategies for losing weight and improving sleep quality is adopting a healthy diet. Reducing the intake of high-calorie, high-fat, and sugary foods while increasing the consumption of fruits, vegetables, whole grains, and lean proteins can help patients lose weight. These dietary changes can also improve sleep by stabilizing blood sugar levels and reducing inflammation, which are often linked to poor sleep quality. The 2018 American Thoracic Society Guideline recommends a reduced-calorie diet, exercise or increased physical activity, and behavioral counseling for weight management .^8^ The diet follows a general recommendation for 1,200-1,500 kcal/day for women and 1,500-1,800 kcal/day for men or reducing caloric intake by 500-1,000 kcal/day.^8^ ^,^ ^22^ In terms of diet composition, the inclusion of patient preferences is usually associated with better adherence. 

There has been a surge of interest in understanding whether diet composition influences OSA severity. The dietary intake of a subset of patients from a randomized, blinded, sham-controlled, multicenter trial of CPAP therapy was analyzed to understand this association.^23^ Interestingly, patients with severe sleep-disordered breathing and a respiratory disturbance index > 50 events/hour had a diet higher in cholesterol, protein, total fat, and total saturated fatty acids than did those with less severe sleep-disordered breathing and those without significant sleep-disordered breathing. 

Recently, a cross-sectional study including 14,210 participants showed that plant-based and vegetarian diets reduced the risk of OSA.^24^ Furthermore, dietary changes to a whole-food, plant-based pattern have been reported to result in lower excessive daytime sleepiness in patients with OSA.^25^ These effects may be mediated by reductions in obesity and low-grade inflammation.^24^ ^,^ ^25^

In a systematic review and meta-analysis including 1,420 patients, lifestyle interventions such as dietary modifications, exercise training, and sleep hygiene significantly reduced the apnea-hypopnea index (AHI), the oxygen desaturation index, and excessive daytime sleepiness, while improving arousal index, sleep efficiency, and rapid eye movement sleep^26^

Recently, in a randomized controlled trial, 89 men with moderate to severe OSA were randomized to receive usual care (CPAP therapy) or an eight-week weight loss and lifestyle intervention involving nutritional behavior change, aerobic exercise, sleep hygiene, and alcohol and tobacco cessation combined with usual care.^27^ The primary outcome was a change in the AHI, with the intervention group having a 51% reduction in the AHI in 8 weeks and a 57% reduction after 6 months. No significant changes occurred in the usual care group. Interestingly, after 8 weeks, 45% of the participants in the intervention group no longer required CPAP therapy, and the results were even better after 6 months, with 61% of the participants no longer requiring CPAP. 

In the longest longitudinal study assessing the impact of lifestyle intervention on OSA severity,^28^ patients were initially randomized for intensive lifestyle intervention focusing on weight loss vs. diabetes support and education. After 10 years, participants in the intensive lifestyle intervention group had reduced OSA severity. Moreover, 34.4% of the participants receiving intensive lifestyle intervention had remission of their OSA in comparison with 22.2% of those on diabetes support and education. Although there was a significant improvement in OSA severity with intensive lifestyle intervention in the ﬁrst 4 years, it was no longer present at the 10-year follow-up.^28^ Such results support the notion that patients require periodic assessment and continued education to maintain the initial results. 

Regular physical activity is effective in reducing OSA symptoms and improving OSA severity. In a meta-analysis including 228 participants, reduced OSA severity (mean difference, −11.4; 95% CI, −13.4 to −9.4 events/hour) and improved quality of life and quality of sleep were reported.^29^ In a more recent meta-analysis, which included 526 participants, exercise training significantly reduced the AHI (unstandardized mean difference = −7.08 events/hour; 95% CI, −9.98 to −4.17; p < 0.00001).^30^ Several mechanisms are implicated in OSA improvement with exercise training, including the following: a reduction of fat deposits around the upper airway and tongue; reduced visceral adiposity and, consequently, improved lung volume; and increased levels of catecholamines that promote visceral fat lipolysis.^31^ Combining exercise with dietary and other therapeutic strategies has yielded the greatest benefits.^31^

Stress management and adequate control of psychiatric symptoms should be included in lifestyle interventions. Secondary analysis of participants in the aforementioned trial showed significant reductions in anxiety and depression scores.^21^ Stress, anxiety, and depression are associated with emotional eating, increased cortisol levels, and disrupted sleep. Incorporating mental health techniques such as mindfulness, meditation, deep breathing exercises, and other relaxation techniques can improve psychiatric symptoms, promote better sleep, and support weight loss. 

## DRUG TREATMENT FOR INDIVIDUALS WITH OBESITY AND OSA

Lifestyle (nutritional and behavioral) interventions typically result in a reduction of 5-10% in weight.^5^ However, lifestyle modifications alone may not result in sustained long-term weight loss. In contrast, pharmacological and surgical interventions may lead to more substantial weight loss and control comorbidities, including OSA.^7^

Despite the global escalation of overweight and obesity, which have reached epidemic proportions,^1^ ^,^ ^32^ affecting more than 50% of the adult population, less than 1% of eligible individuals receive antiobesity medications, and only 0.25% of those eligible are referred for bariatric surgery.^33^ This contrasts with the 86% of patients who are eligible for diabetes pharmacotherapy and who receive appropriate treatment.^33^

Reasons for the undertreatment of obesity include stigma, bias, and a reluctance to accept obesity as a chronic disease on the part of physicians and obese individuals alike.^34^ In a study analyzing barriers to effective treatment of obese individuals, it was reported that although most physicians acknowledge obesity as a chronic disease, relatively few treat it effectively.^34^ Moreover, the widespread blame directed toward obese individuals further exacerbates the barriers to care for this population.^34^

It is well established that even modest reductions in body weight (of approximately 10%) can have a long-term impact on cardiovascular risk and mortality in obese individuals.^35^ Current clinical guidelines recommend that the initial choice of antiobesity medication be defined on the basis of physician/patient preference, potential drug interactions, associated comorbidities, and the risk of adverse events. Unfortunately, the availability of medications approved for the treatment of obesity remains limited, and considerable interindividual variability exists in weight loss response. Notably, achieving weight loss > 5% in the first 3 months of clinical treatment is the only reliable predictor of sustained long-term success. Given the marked heterogeneity in the etiology and clinical manifestations of obesity, as well as the wide variability in response to dietary, pharmacological, and surgical interventions, therapeutic strategies should prioritize individualized therapy and ongoing evaluation of clinical outcomes.^36^

Pharmacological treatment should focus not only on weight reduction but also on improving health parameters (including metabolic parameters, mechanical parameters, mental health, and quality of life); maintaining weight after lifestyle-induced weight loss; and controlling cravings. The indications for the pharmacological treatment of obesity are presented in Chart 1. 

**Chart 1:** Indications for the pharmacological treatment of obesity.

1. Patients with a BMI equal to or greater than 30 kg/m^2^ who have not succeeded in losing weight with nonpharmacological treatment. 2. Patients with a BMI equal to or greater than 27 kg/m^2^ and comorbidities (e.g., type 2 diabetes mellitus, hypertension, dyslipidemia, and obstructive sleep apnea) who have failed to lose weight with nonpharmacological treatment. 3. Individuals with an increased waist circumference (considered viscerally obese) and the presence of comorbidities associated with obesity and metabolic syndrome (37).^37^

Medications currently approved for obesity management include orlistat, phentermine-topiramate (not in Brazil), sibutramine (only in Brazil), naltrexone-bupropion, liraglutide, semaglutide, and tirzepatide. The choice of medication should be individualized on the basis of the following: therapeutic goals; patient values and preferences; comorbidities; mechanisms of action; side effects/tolerability; safety; mode and frequency of administration; and cost.^36^ A clinically meaningful response is typically defined as a 5% weight loss within 12 weeks at the maximum tolerated dose. The currently approved medications, doses, mechanism of action, average weight loss, common side effects, and contraindications are listed in Chart 2.^37^ ^,^ ^38^ The impact of anti-obesity medications on OSA, measured by the AIH, has been tested in some studies, presented in Chart 3.

**Chart 2:** Pharmacological obesity treatment: mechanisms of action, side effects, and expected weight loss.

Medication	Year of approval (FDA)	Dose	Mechanism of action	Side effects	Contraindication/Caution	Mean weight loss in one year
Orlistat	1999	60-120 mg three times a day before meals	Orlistat acts locally as a gastrointestinal tract lipase inhibitor, partially reducing the subsequent absorption of monoacylglycerols and free fatty acids.	Diarrhea, flatulence, abdominal pain, fecal urgency, steatorrhea/incontinence, oily spotting, flatulence, and decreased absorption of fat-soluble vitamins (vitamins A, D, E, and K)	Chronic malabsorption syndrome, cholestasis, history of calcium oxalate kidney stones, pregnancy, breast feeding, cholestasis malabsorption syndromes, warfarin, and antiepileptic drugs	2.9-3.4%
Sibutramine	1997	10-15 mg	Sibutramine is a serotonin and noradrenaline reuptake inhibitor that acts on hypothalamic 5-HT2A, 2B, and 2C receptors, enhancing satiety and stimulating sympathetic activity, leading to increased energy expenditure.^49^ However, increases in sympathetic activity were thought to be the cause of increased nonfatal cardiovascular events in patients with cardiovascular disease in a randomized controlled trial .^50^ Therefore, sibutramine was banned in several countries by the European Medicine Agency and the FDA. In Brazil, the Brazilian Health Regulatory Agency approved the use of sibutramine for patients without cardiovascular risk factors, a B2 prescription being required.	Dry mouth, headache, insomnia, constipation, and irritability	Cardiovascular diseases and uncontrolled hypertension	4.2%
Liraglutide	2014	3 mg dose titration: 0.6 mg/1.2 mg/1.8 mg/2.4 mg/3.0 mg administered subcutaneously once a day	Liraglutide is a GLP-1 analog, with 97% homology to human GLP-1 and agonist action on its receptors. It acts in reducing caloric intake; increasing satiety; enhancing beta-cell function; reducing apoptosis; increasing insulin secretion dependent on glucose intake; reducing inflammation, systolic blood pressure, lipotoxicity, and steatosis; increasing insulin sensitivity; and reducing the rate of gastric emptying)^51^	Nausea, vomiting, diarrhea constipation, fatigue, and rash	Gallbladder disease, pancreatitis, history of medullary thyroid cancer, or MEN 2	9%
Semaglutide	2021	Dose titration: 0.25 mg/0.5 mg/1.0 mg/1.7 mg/2.4 mg administered subcutaneously once a week	Semaglutide is a GLP-1 analog, with a 94% homology to human GLP-1. The amino acid substitution at position 8 prevents degradation by dipeptidyl peptidase-4 and augments its half-life of approximately one week. Semaglutide’s effect on weight loss is through direct activation of the hypothalamus with homeostatic regulation of appetite and direct activation of the rhombencephalon with hedonic regulation of appetite.^52^ It reduces caloric intake, hunger, and frequency of binge eating. It increases satiety and provides better control of eating and weight loss.	Nausea, vomiting, diarrhea, and constipation	Previous pancreatitis, a family history of pancreatic cancer, medullary thyroid carcinoma, or MEN 2, and advanced chronic kidney disease, and diabetic retinopathy	16%
Bupropion and naltrexone	2014	8/90 mg dose titration: 8 mg/90 mg 1 tablet once a day 1 tablet twice a day 2 tablets every morning and 1 tablet every evening and 1 tablet every afternoon/evening, 2 tablets twice a day	Bupropion reduces uptake of dopamine (and norepinephrine), activates proopiomelanocortin neurons, and modulates reward pathways. Naltrexone: An opiate antagonist that blocks the feedback inhibition of β-endorphin. Naltrexone and bupropion act synergistically to activate proopiomelanocortin neurons, resulting in appetite suppression. Also, naltrexone/bupropion synergistically reduce food intake associated with mesolimbic reward pathways.	Nausea, vomiting, constipation, headache, dizziness, and anxiety. Monitor for increasing blood pressure.	Uncontrolled hypertension, seizure disorders, anorexia nervosa, bulimia nervosa, drug or alcohol withdrawal, and monoamine oxidase inhibitors	6%^(44,53)^
Tirzepatide	2023	Dose titration: tirzepatide at 2.5 mg, 5 mg, 10 mg, and 15 mg administered subcutaneously once a week	Tirzepatide is a single synthetic peptide that stimulates GLP-1 and GIP receptors. GLP-1 and GIP are the two known incretin peptides secreted from enteroendocrine cells in the gut mucosa in response to food ingestion. GLP-1, in the brain, decreases appetitive drive and induces fat metabolism and weight loss. The effects of GIP on appetite, fat metabolism, and energy balance are less clear.^54^	Gastrointestinal problems such as nausea, vomiting, constipation, and diarrhea. Tirzepatide slows gastric emptying and raises the concern about risk of aspiration.	Family history of medullary thyroid carcinoma or patients with MEN 2	Up to 96% of participants achieved > 5% weight reduction, with up to 63% of patients achieving ≥ 20% weight reduction.(54)^45^

FDA: U.S. Food and Drug Administration; GLP-1: glucagon-like peptide-1; GIP: gastric inhibitory polypeptide; and MEN 2: multiple endocrine neoplasia, type 2.

**Chart 3:** Effects of pharmacological obesity treatment on weight reduction and the apnea-hypopnea index.

Study	Type of study	Intervention	Follow-up	Population	N	Male %	Age, years Mean ± SD	BMI Mean ± SD	Baseline AHI Mean ± SD	Weight/AHI alterations
Sibutramine										
Yee BJ et al.^55^	Open uncontrolled cohort study	Sibutramine	6 months	Obese patients with OSA	87	100	46.3 ± 9.3	34.2 ± 2.8	46.0 ± 23.1 events/h	Weight changes 8.3 ± 4.7 kg AHI fell by 16.3 ± 19.4 events/h
Liraglutide										
Blackman et al. ^56^	Randomized double-blind trial	Liraglutide at 3 mg	32 weeks	Obese patients with moderate to severe OSA	359	71.9	48.6 ± 9.9	39.1 ± 3.2	49.2 events/h	The rate of weight loss was greater with liraglutide than with placebo (−5.7% vs. −1.6%) AHI changes were greater with liraglutide than with placebo (−12.2 vs. −6.1 events/h)
Tirzepatide										
Malhotra et al.^57^	Randomized double-blind controlled trials	Tirzepatide at 10/15 mg	52 weeks	Trial 1 Moderate to severe OSA Obese men without CPAP	234	68.4 55	47.3 ± 11.0	39.7 ± 7.3	51.5 events per hour	In trial 1, the mean change in AHI at week 52 was −25.3 events per hour with tirzepatide and −5.3 events per hour with placebo. The mean weight change was 18.1 kg in the tirzepatide group vs. 1.3 kg in the placebo group. In trial 2, the mean change in AHI at week 52 was −29.3 events per hour with tirzepatide and −5.5 events per hour with placebo. The mean weight change was 20.1 kg in the tirzepatide group vs 2.3 kg in the placebo group.
Trial 2 Moderate to severe OSA Obese men with CPAP	235	50.8 ± 10.7	38.6 ± 6.1	49.5 events per hour in trial 2						
Orlistat										
Svendsen M et al.^58^	Prospective	Orlistat at 360 mg	1 year	Obese subjects with OSA	63	76	Range, 32-62	34.6 ± 4.7	Not shown	mean weight reduction was -3.5 ± 8.1 kg
Phentermine/topiramate										
Winslow et al.^59^	Randomized, double-blind, placebo-controlled study	Phentermine at 15 mg/topiramate at 92 mg	30 weeks	Obese with Moderate to severe OSA	22	45	53.4 ± 6.95	36 ± 3.07	44.2 ± 22.40	There was a significant weight reduction (−10.2%) and concomitant improvements in OSA (31.5 events/h) parameters in the intervention group.
				Controls	23		51.4 ± 5.74	35.3 ± 3.14	45.2 ± 34.25	

AHI: apnea-hypopnea index; and OSA: obstructive sleep apnea.

## ASSESSING AND OPTIMIZING RESPONSES TO ANTIOBESITY MEDICATIONS

Predicting which medication will be most effective for an individual patient remains challenging. Treatment goals should therefore prioritize outcomes that the patient identifies as important to them, including weight reduction, improvement in health parameters (metabolic parameters, mechanical parameters, mental health, and quality of life), weight maintenance after lifestyle-induced weight loss, and better control of cravings. If treatment goals are not met after 3-6 months of treatment, clinicians should reassess adherence, tolerability, barriers to health-behavior change, and any psychosocial or medical factors that may be limiting progress, and consider adding or substituting medication. This comprehensive approach to pharmacological management highlights the individualized nature of treatment for obesity and its related comorbidities (especially OSA), and emphasizes the importance of a holistic, patient-centered strategy to achieve sustainable health improvements and reduce the overall burden of obesity-related disease. 

## THE IMPACT THAT POSITIVE PRESSURE TREATMENT FOR OSA HAS ON WEIGHT LOSS

On the basis of the premise that reduced sleepiness and improved vitality led to increased energy expenditure, it was previously assumed that treating OSA with CPAP would be sufficient to lead to spontaneous weight loss.^39^ However, recent evidence does not support this assumption. In a randomized study of 146 obese patients with moderate to severe OSA, participants were assigned to one of three groups: treatment with CPAP alone; diet and exercise associated with cognitive-behavioral measures; and CPAP plus the same diet and exercise approaches and behavioral measures.^40^ The study found an average weight loss of 7 kg in the groups that included diet and exercise, whereas, in the CPAP-only group, no weight loss occurred (40).^40^ A meta-analysis of randomized studies in which CPAP was used for OSA treatment reported a weight gain of 0.4 kg in an average period of 3 months after CPAP initiation.^41^ A more recent meta-analysis confirmed that CPAP is associated with increases in BMI. Inadequate adherence to CPAP, with less than 5 h of use per night, was associated with weight gain,^42^ whereas patients who used CPAP for more than 6 h per night had weight loss. 

Although the mechanisms by which CPAP leads to weight gain are unclear, in a study involving patients regularly using CPAP, the withdrawal and reintroduction of CPAP led to a gain of 0.4 kg after 1 week.^43^ The short intervention period and the correlation between body water gain (as detected by bioimpedance) and weight gain suggested that the effect of CPAP on weight gain may be due to water retention. Taken together, these data indicate that CPAP alone is insufficient to induce weight loss. Therefore, the treatment of overweight or obese OSA patients should consider lifestyle changes that include diet and exercise and incorporate CPAP or an alternative form of OSA treatment when indicated. 

## BARIATRIC SURGERY IN OBESE PATIENTS WITH OSA

Bariatric surgery is one of the most efficient therapies for weight loss and is recommended for individuals with class III obesity (a BMI > 40 kg/m^2^) or class II obesity (a BMI = 35-39.9 kg/m^2^) accompanied by comorbidities. Common surgical procedures include adjustable gastric banding, Roux-en-Y gastric bypass (RYGB), and laparoscopic sleeve gastrectomy (LSG). 

The substantial weight loss provided by bariatric surgery improves OSA and is particularly important for obesity hypoventilation syndrome patients, in whom greater weight reduction is necessary to reduce the risk of comorbidities and mortality. However, there is a significant variability in postoperative weight loss and AHI reduction, and many patients continue to require OSA treatment. In a recent systematic review and meta-analysis including 2,310 OSA patients from 32 studies, significant reductions in AHI (weighed mean difference [WMD] = −19.3; 95% CI, −23.9 to −14.6) and respiratory disturbance index (WMD = −33.9; 95% CI, −42.1 to −25.7) were observed after surgery. However, the rate of OSA remission was only 65%.^44^

Results from randomized controlled trials suggest that the impact of bariatric surgery on the BMI and AHI is of a smaller magnitude than that reported in nonrandomized studies.^5^ For instance, in a randomized controlled trial comparing bariatric surgery with a conventional weight loss program, it was found that, despite greater weight reduction with surgery, the between-group difference in AHI was not significant, with a reduction of 11.5 events/hour (95% CI, −28.3 to 5.3 events/hour; p = 0.18).^45^ In a randomized controlled trial comparing gastric banding with intensive nutritional care, the rate of weaning from nocturnal noninvasive ventilation was evaluated after 1 year of treatment and after 3 years of treatment. The rate did not differ significantly between the gastric banding and intensive nutritional care groups at year 1 (35% vs. 13%) or year 3 (14% vs. 21%), even though gastric banding resulted in higher weight loss.^46^ These results reinforce the need for close follow-up after surgery, even when there is significant weight loss. 

Most studies investigating the impact of bariatric surgery on OSA evaluate more severe patients (i.e., patients with class III obesity). In a randomized study following patients with class I or class II obesity for 3 years after RYGB, it was shown that RYGB significantly reduced the severity of OSA when compared with usual care.^47^ At the end of the study, the proportion of patients without OSA increased from 4.2% to 70.8% in the RYGB group, accompanied by reductions in moderate (from 41.7% to 8.3%) and severe OSA (from 20.8% to 0%), whereas, in the usual care group, there was an increased prevalence of moderate OSA. 

In a randomized controlled trial, long-term outcomes of LSG and RYGB were evaluated.^48^ After a 10-year follow-up period, 5 of 31 patients (16%) in the LSG group vs. 9 of 29 (31%) in the RYGB group had discontinued CPAP, and 8 of 31 (26%) vs. 4 of 29 (14%) had reduced CPAP settings. However, 58% in the LSG group and 55% in the RYGB group had no change in CPAP settings. Currently, there is no definitive evidence of superiority of any surgical technique in reducing OSA severity and achieving OSA remission. 

Although bariatric surgery typically reduces OSA severity, significant OSA may persist, especially in patients with class III obesity, those undergoing gastric banding, and those with very severe OSA at baseline. Therefore, it is important to continue follow-up after surgery. Several factors have been implicated in the persistence of OSA, including insufficient weight loss; rebound weight gain; nonanatomical pathophysiological factors (e.g., high loop gain and low arousal threshold); nonadherence to diet and physical activity; and aging.^5^

## FINAL CONSIDERATIONS

Weight management is paramount in the treatment of OSA, given that overweight and obesity are the main modifiable risk factors for the development of OSA. Weight loss not only reduces OSA severity effectively but also contributes to better sleep and quality of life and reduces the risk of cardiovascular and metabolic comorbidities. Emerging pharmacological interventions offer promising new options, and therapeutic success is most likely achieved with individualized combination therapy. 
